# Effects of dietary valine supplementation during late gestation on the reproductive performance and mammary gland development of gilts

**DOI:** 10.1186/s40104-019-0420-z

**Published:** 2020-02-19

**Authors:** Long Che, Mengmeng Xu, Kaiguo Gao, Li Wang, Xuefen Yang, Xiaolu Wen, Hao Xiao, Zongyong Jiang

**Affiliations:** 1 State Key Laboratory of Livestock and Poultry Breeding; Key Laboratory of Animal Nutrition and Feed Science in South China, Ministry of Agriculture; Guangdong Public Laboratory of Animal Breeding and Nutrition; Guangdong Key Laboratory of Animal Breeding and Nutrition, Institute of Animal Science, Guangdong Academy of Agricultural Sciences, No.1 Dafeng Street, Wushan Rd, Tianhe District, Guangzhou, 510640 Guangdong China; 2 College of Animal Science and Technology, Henan University of Animal Husbandry and Economy, Zhengzhou, 450046 Henan China

**Keywords:** Gilt, Mammary gland, Piglet growth, Valine

## Abstract

**Background:**

Mammary gland development during late gestation in gilts is a major factor that alters the composition of colostrum and growth performance of piglets. Plasma valine is taken up and metabolized extensively by the mammary gland; however, the effects of valine on mammary gland development during late gestation are still unclear. Thirty primiparous gilts were divided into three treatment groups (*n* = 10) and received one of the three diets starting on day 75 of gestation until the day of farrowing. The total dietary valine to lysine ratio of the three diets was 0.63 (LV), 0.73 (MV), and 0.93 (HV), respectively.

**Results:**

Dietary valine supplementation during late gestation did not affect (*P* > 0.05) the litter size and weight at farrowing; however, the piglet weight and average daily gain at weaning were linearly increased (*P* < 0.05) as the dietary valine increased. The highest piglet weight at weaning was observed when the gilts were provided the HV diet. Dietary valine supplementation linearly elevated (*P* < 0.05) protein, fat and solids-not-fat and some free amino acids content in colostrum. The concentration of prolactin in plasma of gilts was linearly increased in response to valine supplementation at days 1 and 10 of lactation (*P* < 0.05). Furthermore, with increasing dietary valine allowance, a linear increase (*P* < 0.05) was observed in the area of the lumen of alveolus and the content of DNA, RNA, and total protein in the mammary tissues at day 1 of lactation. Moreover, the protein expression of cyclin D1, p-mTOR, p-S6, and p-4EBP1 was also linearly increased (*P* < 0.05) in the mammary tissue at day 1 of lactation. However, no difference (*P* > 0.05) was observed in the indices related to mammary development and the mTOR signaling pathway at day 21 of lactation.

**Conclusion:**

The results revealed that increasing the total dietary valine to lysine ratio to 0.93 during late gestation significantly enhances the piglet weight and average daily gain at weaning probably due to improved development of mammary gland.

## Background

Colostrum is essential for early postnatal survival and growth, partially by providing energy for thermoregulation and immunoglobulins for passive immunity and by stimulating intestinal growth and maturation [1]. It is known that the crucial factors determining the capacity of the mammary gland to synthesize colostrum largely depend on the development of mammary gland, especially during gestation [2]. A period of extensive mammary gland development in gilts begins in the third trimester of gestation [3]. It has been shown that the crude protein content per gland of the mammary tissue increases by 0.14 g/d from day 0 to 80 of gestation and 3.41 g/d from day 70 of gestation suggesting that it is a critical window of mammary gland development [4]. Therefore, an optimal nutritional strategy during this period may provide an effective method to ensure the physiological needs of mammary growth and maximal growth of offspring. Studies have highlighted that increasing the supplementation of dietary amino acids during late gestation can improve the lactation performance of sows and the growth of nursing piglets, for example lysine [5] and arginine [6]. However, only a few studies have investigated the effects of dietary valine supplementation during late gestation on the subsequent reproductive performance.

Valine is considered the second- or third-most limiting amino acid for lactating sows after lysine or threonine [7, 8]. Meanwhile, valine is transported at the highest dose (valine > leucine > isoleucine> threonine > arginine > lysine) in the mammary gland when sows are provided standard commercial diets [9], suggesting that valine may play a crucial role in physiological metabolism in the mammary gland. Notably, recent *in vivo* studies provide strong evidence that valine could increase cell proliferation and protein synthesis in porcine mammary gland epithelial cells via the activation of the mammalian target of rapamycin (mTOR) signaling pathway [10, 11]. Therefore, we hypothesized that the development of mammary gland and performance of gilts can be increased by increasing dietary valine supplementation during late gestation.

## Materials and methods

All experimental procedures followed the current law regarding animal protection (Ethic Approval Code: 5YXK2016–0165) and were approved by the Guide for the Care and Use of Laboratory Animals prepared by the Animal Care and Use Committee of Guangdong Academy of Agricultural Sciences.

### Animals and dietary treatments

The study was conducted in Guangdong Academy of Agricultural Sciences from August 2018 to October 2018 using 30 pregnant gilts (Yorkshire × Landrace). All gilts were synchronized for estrus and artificially inseminated. The drugs used for synchronization of estrus, including altrenogest, pregnant mare serum gonadotropin (PMSG), and gonadotropin-releasing hormone (GnRH) were obtained from Sansheng Biological Technology (Ningbo, Zhejiang, China). All gilts were housed individually and fed the same diet including 3.09 Mcal/kg digestible energy (DE) and 12.95% crude protein (CP) at 2.08 kg/d from day 0 to 75 of gestation. On day 75, the bodyweight and backfat thickness were measured, and the gilts were randomly allocated to one of the three nutritional groups with 10 replicates per group: low-valine (LV), medium-valine (MV), and high-valine (HV) groups. The gestation basal diet (Table 1) was formulated to contain 0.85% total lysine, with all amino acids other than valine formulated to meet requirement as recommended by National Research Council (2012) [12], and the total valine to lysine ratio in the basal diet was 0.63 (LV group). The basal diet was supplemented with valine to provide additional treatments (MV and HV groups) with the ratio of total valine to lysine were 0.73 and 0.93, respectively. These gilts in the LV, MV and HV groups were fed the diet with the ratio of total valine to lysine were 0.63, 0.73 and 0.93, respectively. The diet in the MV group represents normal valine requirement of gestation gilts recommended by NRC (2012) [12]. Valine was supplemented with crystalline *L*-valine of 98% purity (CJ Shenyang Biotech Co., Ltd., Liaoning, China). The isonitrogenous diet of the LV group was supplemented with 0.25% alanine and MV diet was supplemented with 0.18% alanine (Table 1). The experimental diets were provided from day 75 of gestation until farrowing on day 0 of lactation.

**Table 1:** Ingredients and nutrient content of diets (as fed basis)

Ingredients	Proportion, %
Corn	71.44
Peanut meal (exp.), 45% crude protein	12.00
Soybean hulls	12.00
Lys·HCl (78.8%)	0.52
Met (98.5%)	0.05
Trp (98%)	0.05
Thr (98%)	0.25
Salt	0.50
Limestone	1.35
Dicalcium phosphate	1.55
Choline chloride	0.15
Vitamin and mineral premix ^a^	0.24
Chemical composition	
Digestible energy (DE) ^c^, MJ/kg	13.05
Crude protein (CP) ^b^, %	13.60
Lysine ^b^, %	0.85
Met + Cys ^b^, %	0.45
Val ^b^, %	0.54
Leu ^b^, %	0.88
Ile ^b^, %	0.30
Ca ^c^, %	0.92
Av. P ^c^, %	0.38

^a^ Provided per kg of diet: 9600 IU vitamin A; 1500 IU vitamin D_3_; 52.2 IU vitamin E, 2.9 mg vitamin K_3_; 1.5 mg vitamin B_1_; 6.02 mg vitamin B_2_; 5.25 mg vitamin B_6_; 0.02 mg vitamin B_12_; 21.5 mg niacin; 30 mg calcium pantothenate; 3.42 mg folic acid; 0.45 mg biotin; 48 mg manganese (MnSO_4_); 132 mg iron (FeSO_4_); 120 mg zinc (ZnSO_4_); 12 mg copper (CuSO_4_); 0.6 mg iodine (CaI_2_O_6_) and 0.3 mg selenium (Na_2_SeO_3_)

^b^ Analyzed values

^c^ Calculated values

### Feeding and management

All gilts were housed in individual feeding stalls and were allowed to consume water *ad libitum*. The mean room temperature was 32.3 °C ± 0.33 °C, 31.8 °C ± 0.31 °C and 30.4 °C ± 0.34 °C in August, September, and October, respectively. The gestation diet contained 3.12 Mcal/kg DE and 13.6% CP, and the lactation diet contained 3.32 Mcal/kg DE and 18.5% CP (on as-fed basis). According to the NRC (2012) [12], the feed intake of all gestating gilts was calculated to be 2.32 kg/d from day 75 to 90 of gestation and 2.72 kg/d from day 91 to 114 regardless of treatments. The gilts received two equal feed allotments at 08:00 and 17:00 h, which was based on the feeding standards for gestating gilts [12]. For lactating gilts, the same diet was provided three times daily and restricted, increasing the amount gradually until day 5 of lactation, after which the diet was provided *ad libitum*. Water was available *ad libitum* throughout the study. On day 107 of gestation, the gilts were moved into farrowing accommodations and housed in individual farrowing crates. Within 24 h of farrowing, the litter size was standardized to 10 piglets per gilt, depending on their availability and body weight, by cross fostering the piglets within the same treatment [13]. The method of cross fostering followed two major principles. Firstly, the piglets with birth weight less than 0.8 kg were eliminated. Secondly, to ensure that the fostered piglets can compete with other piglets, the piglets were transferred usually from the first farrowing with higher weight to later farrowing. When there was piglet mortality, the dead piglets were replaced by another piglet with similar bodyweight and age. All piglets were injected iron and had their needle teeth clipped, and the males were surgically castrated on day 3 or 4 postpartum. The piglets were weaned on day 21 of lactation.

### Collection of reproductive performance

The gilts were weighed at day 75 of gestation and days 0 and 21 of lactation. P2 backfat thickness (6.5 cm from the midline over the last rib) was measured at days 0 and 21 (weaning) of lactation by A-mode ultrasonography (Lean-meater; Renco Corporation, Minneapolis, MN, USA). The daily feed intake of the gilts was recorded to calculate the total and average daily feed intake (ADFI). On the day of farrowing, the number of piglets born live (litter size), individual piglet weight and litter weight were recorded. After cross-fostering, individual weight and litter weight were recorded. During lactation, the piglets were individually weighed at weaning. According to the weight of piglets, the average daily gain (ADG) and litter weight gain during lactation were calculated. In addition, one piglet from each group was slaughtered for another study at day 7 of lactation.

### Collection of blood and milk

Blood samples of gilts were collected in heparinized tubes via the ear vein before feeding on the morning of days 0 and 10 of lactation. All blood samples were kept on the ice for about 10 min and then centrifuged at 3000×*g* for 15 min at 4 °C. Plasma was obtained from the supernatant and stored at − 20 °C for downstream analysis. Milk samples were collected and pooled from all the functional glands on the left side of the gilt after thoroughly cleaning the udder. After parturition, a colostrum sample (30 mL) from each gilt was collected by hand-milking before any piglet suckled the mammary teats. A milk sample (30 mL) from each gilt was collected at day 10 of lactation by injecting 1 mL (10 IU) of oxytocin (Sansheng Biological Technology, Ningbo, Zhejiang, China) via ear venipuncture to stimulate milk release. The colostrum and milk samples were stored at − 20 °C until analysis.

### Collection of mammary tissue

The mammary tissues of each group (*n* = 4) were collected randomly from the third mammary gland on the left side of the body at days 1 and 21 of lactation. The mammary tissues were surgically obtained according to previously published methods [14], with slight modifications. Firstly, the gilts were deeply anesthetized with Zoletil 50 (composed of tiletamine hydrochloride and zolazepam; Zoletil 50 Vet, Virbac, France) at a dose of 0.1 mg/kg of body weight, administered through intramuscular injection. The incision area of the mammary gland was then anesthetized with a subcutaneous injection of lidocaine (1 mL, 2%). Finally, a 2-cm incision was made at 5 cm dorsal to the perimeter of the nipple areola and three pieces of mammary tissue were collected for future studies. The incision was stitched after disinfection. The piglets began to nurse normally after the gilts’ full recovery from anesthesia.

### Mammary tissue morphology

To observe the morphology of mammary tissues and measure the area of lumen of alveolus, mammary tissue histological sections were analyzed according to Xu et al. [15]. Four percent paraformaldehyde was used to fix mammary tissues, which were dehydrated and embedded in paraffin. The samples were cut into 4-μm slices using a microtome. The slices were then stained with hematoxylin and eosin after decoloration with dimethylbenzene and dewaxed with ethanol. Finally, the color images were taken using a bright field microscope. The area of lumen of alveolus was measured using the software Computer Aided Drafting (CAD) system.

### Chemical analyses

Free amino acid composition in the plasma and milk and hydrolytic amino acid composition in diets were analyzed using an amino acid analyzer (L-8900; Hitachi, Tokyo, Japan). Total tissue DNA and RNA were isolated from the mammary tissue using the HiPure Tissue DNA Mini Kit (Magen, Guangzhou, China) and TRIzol (Invitrogen, Carlsbad, CA, USA) respectively, according to the manufacturer’s instructions. The concentration of DNA and RNA was determined using Nanodrop ND-1000 (Nanodrop Technologies, DE, USA). The concentration of protein was determined using the BCA Protein Assay Kit (Thermo Scientific, Waltham, MA, USA) after extraction.

The concentrations of glucose, triglyceride, cholesterol, urea nitrogen, and total protein in the plasma was measured using the respective commercial kits (Nanjing Jiancheng Bioengineering Institute, Nanjing, China), following the manufacturer’s instructions. The concentration of prolactin and insulin in the plasma was measured in accordance with the manufacturer’s protocols of the pig ELISA kits (CUSABIO Biotech Co., Ltd., Wuhan, China).

The expression levels of the proteins were determined by western blotting, as previously described [16]. For extraction of total proteins, frozen mammary tissues were crushed and homogenized with RIPA buffer (Beyotime, Shanghai, China). The tissue lysates were centrifuged at 12,000×*g* for 15 min at 4 °C, and the supernatant fluid was used for determination of protein concentration using BCA protein assay kit (Thermo Scientific, MA, USA). Samples containing the same amount of protein (50 μg) were separated by SDS-PAGE and then transferred onto polyvinylidene difluoride membranes. After blocking, membranes were incubated with a primary antibody overnight and then the membranes were incubated secondary antibody. Anti-mTOR (#2983 s), anti-phospho-mTOR (#2971S), anti-S6 (#2317), anti-phospho-S6 (#4858), anti-4EBP1 (#9644), anti-phospho-4EBP1 (#2855) and anti-mouse IgG (#7076) antibodies were obtained from Cell Signaling Technology (Beverly, MA, USA). Anti-phospho-Stat3 (ab76315), anti-phospho-stat5 (ab32364), and anti-rabbit IgG (ab6721) antibodies were obtained from Abcam (Cambridge, MA, USA). Anti-cyclin D1 (sc8396) and anti-β-casein (sc166684) antibodies were obtained from Santa Cruz (Dallas, Texas, USA). Anti-β-actin (anm40032) antibody was obtained from Amyjet Scientific (Abbkine, Wuhan, China). Immunoreactivity was visualized using a chemiluminescent HRP substrate (Millipore, MA, USA) and a VersaDoc imaging system (Bio-Rad, CA, USA). Band densities were determined using Image J software and expressed relative to that of β-actin.

### Statistical analysis

Data were analyzed as a completely randomized design using the PROC MIXED procedure of SAS statistical software program (version 9.4, SAS Institute Inc., Cary, NC, USA), with individual gilt and litter considered as the experimental unit. Litter size at birth was used as the covariate for the average birth weight and litter weight, and body weight after cross-fostering was used as the covariate for the weight and litter weight during lactation. Orthogonal polynomial contrasts were used to determine the linear and quadratic effects of dietary valine supplementation on the response variables, and Tukey test was used to determine the differences among the groups. All the experimental data are presented as mean ± SEM. The results with a probability value of < 0.05 were considered statistically significant, whereas those with a probability value of 0.05 ≤ *P* < 0.10 were considered a tendency.

## Results

### Gilt and piglet performance

The changes in the body weight, and back fat thickness of gilts from day 75 to farrowing, and from farrowing to weaning, were not affected (*P* > 0.05) among the different treatment groups (Table 2). The feed intake of gilts in the HV group was significantly increased (*P* < 0.05) than that in the MV group between days 0 and 7, but there was no significant difference (*P* > 0.05) when compared with the LV group. In addition, there was no significant difference in feed intake from day 8 to 14 and from day 15 to 21 among the treatment groups (*P* > 0.05) (Table 2).

**Table 2:** Effect of dietary valine: lysine ratio during late gestation on gilt’s performance

Items	Dietary treatments			Linear	Quadratic
	LV	MV	HV		
Body weight, kg					
Day 75 of gestation, kg	188.81 ± 6.57	189.17 ± 4.54	189.00 ± 4.64	0.909	0.610
Day 0 of lactation, kg	196.81 ± 7.17	194.33 ± 5.66	192.25 ± 4.56	0.689	0.468
Day 21 of lactation, kg	186.56 ± 6.71	186.39 ± 5.41	185.19 ± 3.98	0.938	0.591
Loss (d 0 - d 21), kg	10.25 ± 2.49	7.94 ± 2.30	7.06 ± 2.56	0.663	0.621
Backfat, mm					
Day 75 of gestation, mm	17.50 ± 1.05	17.09 ± 0.90	19.50 ± 1.05	0.139	0.376
Day 0 of lactation, mm	19.12 ± 1.05	18.09 ± 0.90	20.00 ± 1.05	0.433	0.276
Day 21 of lactation, mm	16.12 ± 0.83	15.91 ± 0.70	16.25 ± 0.83	0.876	0.786
Loss (d 0- d 21), mm	3.00 ± 0.78	2.18 ± 0.67	3.75 ± 0.78	0.374	0.239
Feed intake, kg/d					
Day 1-7 of lactation, kg/d	3.04 ± 0.18^ab^	2.81 ± 0.17^b^	3.51 ± 0.20^a^	0.049	0.091
Day 7-14 of lactation, kg/d	5.08 ± 0.34	5.13 ± 0.32	5.53 ± 0.34	0.334	0.811
Day 14-21 of lactation, kg/d	5.95 ± 0.26	5.46 ± 0.23	6.15 ± 0.26	0.386	0.082
Day 1-21 of lactation, kg/d	4.57 ± 0.24	4.37 ± 0.21	4.86 ± 0.24	0.290	0.291

Results were presented as mean values with their standard errors, *n* = 10. LV: total valine: lysine = 0.63: 1; MV: total valine: lysine = 0.73: 1; HV: total valine: lysine = 0.93: 1. Means not sharing the same letter are different (*P* < 0.05)

The number of piglets born alive, average individual weight and litter weight at birth were not different (*P* > 0.05) among the treatment groups (Table 3). Dietary valine supplementation during late gestation did not affect the litter size at weaning; however, the piglet weight at weaning and the average daily gain at day 21 linearly increased (*P* <  0.05) with increasing dietary valine allowance (Table 3).

**Table 3:** Effect of dietary valine: lysine ratio during late gestation on piglet’s performance

Items	Dietary treatments			Linear	Quadratic
	LV	MV	HV		
Litter size at birth (live)	11.37 ± 0.42	11.25 ± 0.49	10.38 ± 0.32	0.206	0.497
Piglet weight at birth, kg	1.26 ± 0.07	1.24 ± 1.00	1.36 ± 0.08	0.438	0.611
Litter weigh at birth, kg	12.50 ± 0.60	13.19 ± 0.88	13.76 ± 0.78	0.517	0.891
Piglet weight after adjust^*^, kg	1.28 ± 0.05	1.26 ± 0.08	1.30 ± 0.05	0.884	0.586
Litter weight after adjust, kg	12.68 ± 0.57	12.62 ± 0.75	13.16 ± 0.62	0.817	0.637
Litter size at weaning	9.00 ± 0.27	9.25 ± 0.16	9.00 ± 0.19	0.810	0.489
Piglet weight at weaning, kg	5.24 ± 0.13^b^	5.33 ± 0.13^b^	5.86 ± 0.19^a^	0.003	0.877
Litter weight at weaning, kg	46.57 ± 1.66^b^	48.20 ± 1.94^b^	52.26 ± 1.75^a^	0.003	0.868
Litter weight gain at weaning, kg	33.62 ± 1.51^b^	34.73 ± 1.13^ab^	38.75 ± 1.32^a^	0.004	0.689
Average daily gain during lactation, g	189.64 ± 5.17^b^	195.61 ± 5.51 ^b^	214.93 ± 8.30^a^	0.008	0.763

Results were presented as mean values with their standard errors, *n* = 10. LV: total valine: lysine = 0.63: 1; MV: total valine: lysine = 0.73: 1; HV: total valine: lysine = 0.93: 1. * adjust: within 24 h of farrowing, the litter size was standardized to 10 piglets per gilt, depending on their availability and bodyweight, by cross fostering the piglets within the same treatment. Means not sharing the same letter are different (*P* < 0.05)

### Milk composition of gilts

In colostrum, a linear increased (*P* < 0.05) in protein, fat, and solids-not-fat content was observed as dietary valine increased. The highest protein, fat, and solids-not-fat content was observed when gilts were provided the HV diet. However, there was no difference (*P* > 0.05) in protein, fat, lactose and solids-not-fat content among the three treatment groups at day 10 of gestation (Table 4). In terms of amino acid composition, dietary valine linearly increased (*P* < 0.01) valine concentration in the plasma at day 0 of lactation; however, the concentration of other amino acids was not different at either day 0 or 10 of lactation (Table 5). Regarding the amino acid composition in colostrum, dietary valine linearly increased (*P* < 0.01) the concentration of valine, glutamate, arginine, aspartate, leucine, lysine, phenylalanine and threonine. As dietary valine increased, there was no difference in the concentration of almost all amino acids except cysteine and glycine in the milk at day 10 of lactation (Table 6). The concentration of glycine in milk was increased (*P* < 0.05) in the HV group than that in the MV and LV group. In addition, the highest cysteine concentration was observed when gilts were provided the LV diet.

**Table 4:** Effects of dietary valine: lysine ratio during late gestation on composition of colostrum and milk

Items	Dietary treatments			Linear	Quadratic
	LV	MV	HV		
Day 1 of lactation					
Protein, %	11.07 ± 0.29^b^	11.47 ± 0.18^b^	12.80 ± 0.20^a^	< 0.001	0.495
Fat, %	3.17 ± 0.14^b^	4.17 ± 0.39^b^	6.23 ± 1.01^a^	0.013	0.978
Lactose, %	3.64 ± 0.05	3.52 ± 0.07	3.50 ± 0.20	0.444	0.872
Solids-not-fat, %	17.98 ± 0.74^b^	19.57 ± 0.29^b^	21.79 ± 0.34^a^	0.002	0.623
Day 10 of lactation					
Protein, %	4.67 ± 0.07	4.53 ± 0.09	4.53 ± 0.03	0.263	0.326
Fat, %	5.93 ± 0.20	6.70 ± 0.46	5.93 ± 0.23	0.724	0.102
Lactose, %	6.80 ± 0.10	6.60 ± 0.12	6.57 ± 0.07	0.172	0.348
Solids-not-fat, %	12.70 ± 0.20	12.47 ± 0.23	12.40 ± 0.15	0.362	0.608

Results were presented as mean values with their standard errors, *n* = 10. LV: total valine: lysine = 0.63: 1; MV: total valine: lysine = 0.73: 1; HV: total valine: lysine = 0.93: 1. Means not sharing the same letter are different (*P* < 0.05)

**Table 5:** Effect of dietary valine : lysine ratio during late gestation on free amino acids in plasma

Amino acids, μg/mL	Day 0 of lactation					Day 10 of lactation				
	Dietary treatments			Linear	Quadratic	Dietary treatments			Linear	Quadratic
	LV	MV	HV			LV	MV	HV		
Val	18.88 ± 0.45^c^	20.87 ± 0.20^b^	30.10 ± 0.05^a^	< 0.001	0.754	24.88 ± 1.28	27.50 ± 1.11	26.58 ± 0.17	0.3182	0.095
Leu	15.86 ± 0.07	15.85 ± 1.47	17.70 ± 0.45	0.159	0.591	25.72 ± 0.78	25.17 ± 0.14	23.81 ± 0.61	0.057	0.475
Ile	9.52 ± 0.59	7.61 ± 0.16	8.94 ± 0.95	0.830	0.080	12.28 ± 0.81	13.38 ± 1.32	10.63 ± 0.53	0.178	0.210
Lys	20.71 ± 0.24	14.66 ± 2.12	17.52 ± 1.65	0.364	0.042	23.45 ± 4.96	29.04 ± 2.48	23.52 ± 1.99	0.820	0.237
Met	4.99 ± 0.44	5.60 ± 0.44	4.49 ± 0.05	0.230	0.134	6.93 ± 0.41	6.75 ± 0.09	6.61 ± 0.51	0.580	0.887
Cys	2.90 ± 0.75	3.21 ± 0.01	3.22 ± 0.33	0.690	0.737	2.50 ± 0.78	2.63 ± 0.39	3.30 ± 0.24	0.298	0.846
Phe	15.12 ± 0.14	13.87 ± 1.28	17.00 ± 0.95	0.126	0.154	21.01 ± 1.29	18.98 ± 0.77	18.53 ± 0.07	0.113	0.308
Thr	31.41 ± 4.86	31.28 ± 4.82	29.02 ± 1.45	0.668	0.899	19.98 ± 1.94	22.84 ± 1.47	22.18 ± 2.74	0.575	0.451
Asp	1.34 ± 0.05	1.85 ± 0.38	1.81 ± 0.06	0.259	0.257	2.36 ± 0.22	1.83 ± 0.02	1.80 ± 0.11	0.047	0.097
Glu	25.39 ± 3.86	28.11 ± 5.41	28.47 ± 1.26	0.634	0.741	23.48 ± 0.85	20.90 ± 1.44	22.44 ± 1.25	0.715	0.079
Arg	22.20 ± 0.94	21.87 ± 1.90	22.16 ± 0.45	0.988	0.844	49.86 ± 4.65	43.48 ± 0.64	40.79 ± 0.95	0.073	0.369
Ala	72.22 ± 7.56	72.79 ± 2.13	72.62 ± 4.73	0.968	0.949	43.80 ± 4.78	47.48 ± 2.35	43.87 ± 2.79	0.886	0.430
Ser	10.85 ± 0.45	9.71 ± 1.00	11.57 ± 0.11	0.293	0.132	14.55 ± 0.37	12.65 ± 1.00	13.43 ± 1.14	0.549	0.223
Gly	41.66 ± 5.65	40.99 ± 1.42	43.16 ± 1.44	0.729	0.795	73.34 ± 3.67	65.20 ± 1.09	65.48 ± 6.64	0.319	0.356
His	12.32 ± 1.22	11.00 ± 0.49	13.34 ± 0.64	0.284	0.165	19.09 ± 0.33	17.07 ± 0.61	16.82 ± 0.69	0.044	0.125

Results were presented as mean values with their standard errors, *n* = 10. LV: total valine : lysine= 0.63 : 1; MV: total valine : lysine = 0.73 : 1; HV: total valine : lysine = 0.93 : 1. Means not sharing the same letter are different (*P* < 0.05)

**Table 6:** Effect of dietary valine : lysine ratio during late gestation on free amino acid in colostrum and milk

Amino acids, μg/mL	Day 0 of lactation					Day 10 of lactation				
	Dietary treatments			Linear	Quadratic	Dietary treatments			Linear	Quadratic
	LV	MV	HV			LV	MV	HV		
Val	7.74 ± 0.38 ^b^	8.16 ± 0.58 ^b^	10.56 ± 0.22 ^a^	0.002	0.364	1.88 ± 0.05	2.00 ± 0.01	1.95 ± 0.04	0.146	0.055
Leu	7.11 ± 0.88 ^b^	11.09 ± 2.98 ^ab^	17.68 ± 1.96 ^a^	0.012	0.868	2.61 ± 0.17	2.48 ± 0.12	2.49 ± 0.27	0.748	0.730
Ile	4.10 ± 0.83	4.75 ± 0.46	5.35 ± 0.04	0.166	0.748	0.66 ± 0.04	0.55 ± 0.10	0.51 ± 0.01	0.252	0.532
Lys	7.89 ± 0.63 ^b^	11.09 ± 0.22 ^a^	12.28 ± 0.33 ^a^	< 0.001	0.018	3.52 ± 0.18	3.59 ± 0.09	3.40 ± 0.14	0.764	0.430
Met	2.54 ± 0.17	2.55 ± 0.06	2.71 ± 0.02	0.259	0.739	1.03 ± 0.04	1.00 ± 0.03	0.96 ± 0.02	0.803	0.881
Cys	1.81 ± 0.27	2.00 ± 0.39	1.91 ± 0.26	0.578	0.130	5.55 ± 0.16 ^a^	3.94 ± 0.07 ^c^	4.45 ± 0.04 ^b^	0.001	< 0.001
Phe	4.74 ± 0.95 ^b^	9.74 ± 3.46 ^a^	9.89 ± 2.18 ^a^	< 0.001	< 0.001	3.74 ± 0.13	2.89 ± 0.06	5.10 ± 1.30	0.168	0.212
Thr	5.98 ± 0.21^b^	6.70 ± 0.30 ^b^	8.74 ± 0.68 ^a^	< 0.001	0.182	11.06 ± 0.14	11.43 ± 0.12	10.96 ± 0.05	0.076	0.083
Asp	13.99 ± 0.98 ^b^	12.36 ± 0.79 ^b^	17.14 ± 0.91 ^a^	0.010	0.233	46.85 ± 0.52	46.37 ± 0.87	44.10 ± 0.72	0.571	0.671
Glu	31.17 ± 2.28	21.69 ± 0.99	35.86 ± 5.23	0.060	0.005	52.14 ± 0.38	52.29 ± 0.76	54.10 ± 0.20	0.438	0.106
Arg	9.24 ± 0.60 ^b^	11.51 ± 0.59 ^b^	15.61 ± 1.77 ^a^	0.018	0.968	9.10 ± 1.64	10.94 ± 0.02	10.52 ± 0.22	0.429	0.298
Ala	12.73 ± 0.98	7.99 ± 1.72	10.09 ± 1.32	0.277	0.039	25.41 ± 0.29	24.11 ± 0.85	24.17 ± 0.17	0.226	0.568
Ser	10.23 ± 0.00	13.31 ± 2.82	15.11 ± 1.78	0.269	0.472	6.28 ± 0.29	5.46 ± 0.24	6.24 ± 0.09	0.071	0.253
Gly	7.87 ± 1.21	15.30 ± 3.93	9.97 ± 0.04	0.153	0.628	24.29 ± 0.21 ^b^	24.20 ± 0.74 ^b^	26.39 ± 0.26 ^a^	0.026	0.776
His	6.14 ± 1.41	8.72 ± 1.68	5.73 ± 1.18	0.346	0.463	4.62 ± 0.11	4.23 ± 0.53	4.24 ± 0.11	0.635	0.819

Results were presented as mean values with their standard errors, *n* = 10. LV: total valine : lysine= 0.63 : 1; MV: total valine : lysine = 0.73 : 1; HV: total valine : lysine = 0.93 : 1. Means not sharing the same letter are different (*P* < 0.05)

### Plasma biochemicals of gilts

There was no significant difference (*P* > 0.05) among the treatment groups in the plasma levels of glucose, urea nitrogen and cholesterol at days 0 and 10 of lactation (Table 7). As dietary valine increased, the concentration of plasma triglyceride and prolactin linearly increased (*P* <  0.05) at day 0 of gestation. In addition, with increasing dietary valine allowance, the concentration of total protein and prolactin in the plasma increased linearly and quadratically (*P* <  0.05) at day 10 of lactation (Table 7).

**Table 7:** Effects of dietary valine: lysine ratio during late gestation on plasma biochemicals

Items	Dietary treatments			Linear	Quadratic
	LV	MV	HV		
Day 0 of lactation					
Glucose, mmol/L	4.92 ± 0.18	5.05 ± 0.17	4.98 ± 0.19	0.870	0.627
Urea nitrogen, mmol/L	2.77 ± 0.17	2.77 ± 0.16	2.50 ± 0.17	0.076	0.539
Triglyceride, mmol/L	0.51 ± 0.11^b^	0.89 ± 0.17^a^	1.11 ± 0.07^a^	< 0.001	0.104
Total protein, g/L	67.36 ± 1.57	70.93 ± 1.01	69.48 ± 1.37	0.448	0.104
Cholesterol, mmol/L	1.64 ± 0.12	1.81 ± 0.08	1.76 ± 0.18	0.487	0.269
Insulin, mIU/L	11.12 ± 0.74	10.47 ± 0.28	11.33 ± 0.79	0.267	0.222
Prolactin, ng/mL	56.54 ± 0.54^c^	64.02 ± 0.89^b^	69.20 ± 0.62^a^	< 0.001	0.026
Day 10 of lactation					
Glucose, mmol/L	5.57 ± 0.13	5.25 ± 0.15	5.25 ± 0.22	0.238	0.324
Urea nitrogen, mmol/L	4.37 ± 0.23	3.98 ± 0.14	3.92 ± 0.26	0.063	0.213
Triglyceride, mmol/L	0.86 ± 0.07^b^	1.07 ± 0.09^a^	1.03 ± 0.08^a^	0.1096	0.046
Total protein, g/L	53.05 ± 1.82^b^	54.55 ± 1.67^b^	72.71 ± 0.83^a^	< 0.001	0.017
Cholesterol, mmol/L	2.71 ± 0.12	2.54 ± 0.26	2.61 ± 0.10	0.694	0.408
Insulin, mIU/L	4.51 ± 0.52	4.95 ± 0.52	4.83 ± 0.46	0.756	0.777
Prolactin, ng/mL	21.65 ± 0.78^b^	22.37 ± 0.16^ab^	23.72 ± 0.57^a^	0.045	0.246

Results were presented as mean values with their standard errors, *n* = 10. LV: total valine: lysine = 0.63: 1; MV: total valine: lysine = 0.73: 1; HV: total valine: lysine = 0.93: 1. Means not sharing the same letter are different (*P* < 0.05)

### Mammary gland development

**Fig. 1:**
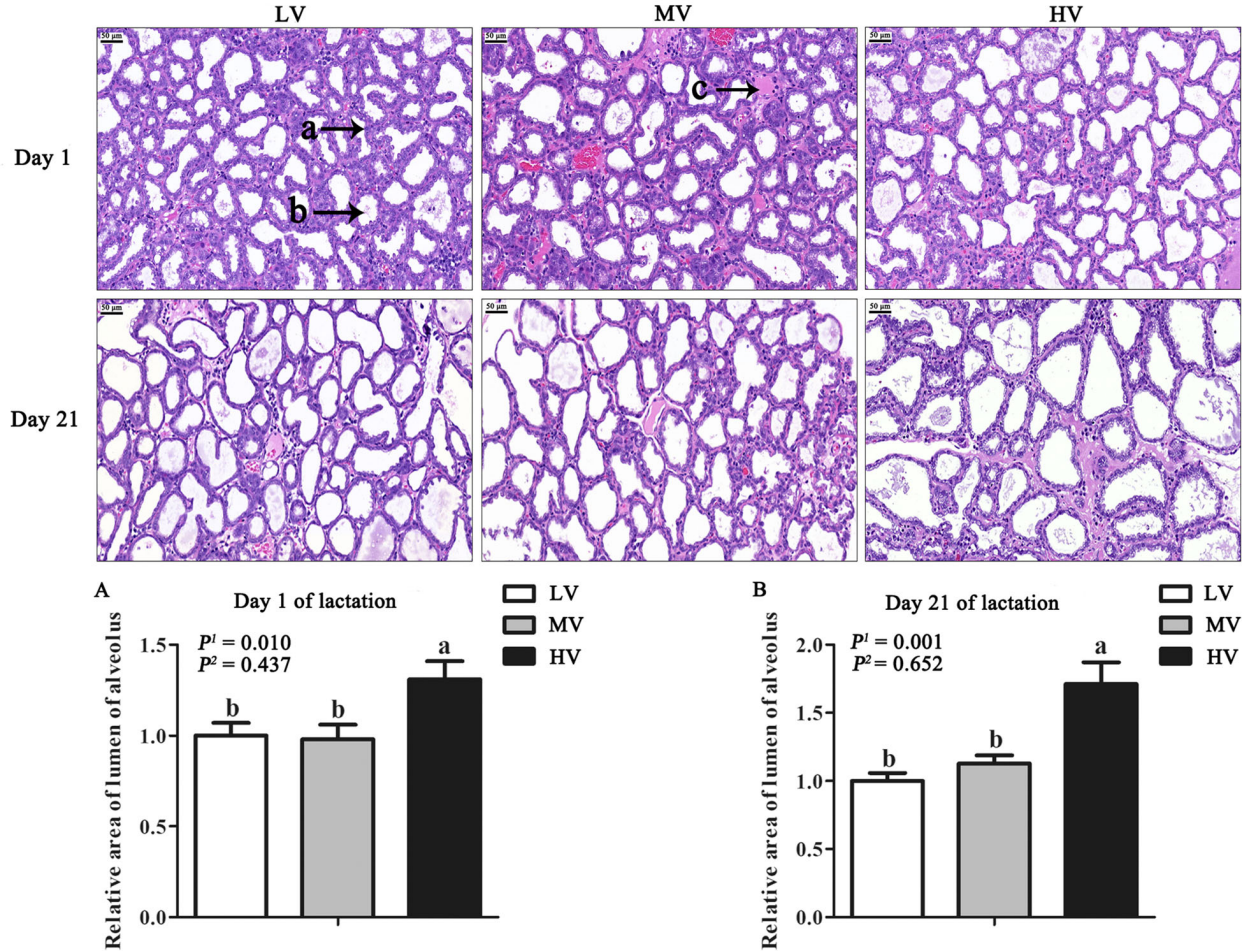
Effects of dietary valine to lysine ratio during late gestation on the morphological characteristics of mammary tissue. LV: total valine: lysine = 0.63: 1; MV: total valine: lysine = 0.73: 1; HV: total valine: lysine = 0.93: 1. (**a**, **b**) shows the area of the lumen of alveolus of the mammary tissue of gilts at days 1 and 21 of lactation, *n* = 4. Scale bars: 400 μm. a: mammary epithelial cells; b: lumen alveolus; c: connective tissue tract

**Fig. 2:**
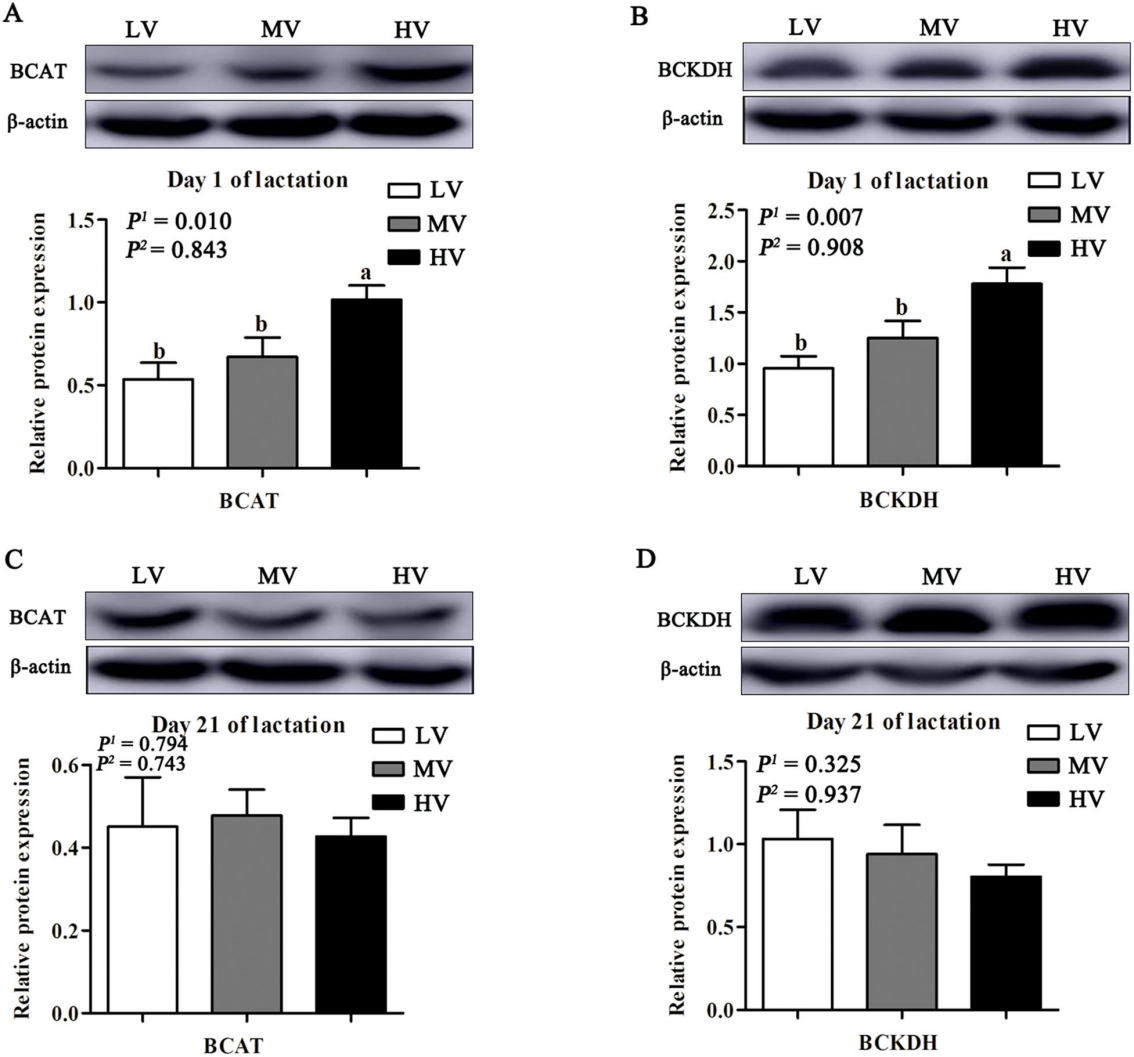
Effects of dietary valine to lysine ratio during late gestation on regulation of valine metabolic enzymes. LV: total valine: lysine = 0.63: 1; MV: total valine: lysine = 0.73: 1; HV: total valine: lysine = 0.93: 1. **a**, **b** shows the relative protein expression of BCAT and BCKDH in mammary tissue of gilts at day 1 of lactation, *n* = 4. **c**, **d** shows the relative protein expression of BCAT and BCKDH in mammary tissue of gilts at day 21 of lactation, *n* = 4. BCAT: branched-chain aminotransferase; BCKDH: branched-chain α-keto acid dehydrogenase. All data with error bars represent the mean ± SEM. *P*^*1*^ = linear, *P*^*2*^ = quadratic. Means not sharing the same letter are different (*P* < 0.05)

**Fig. 3:**
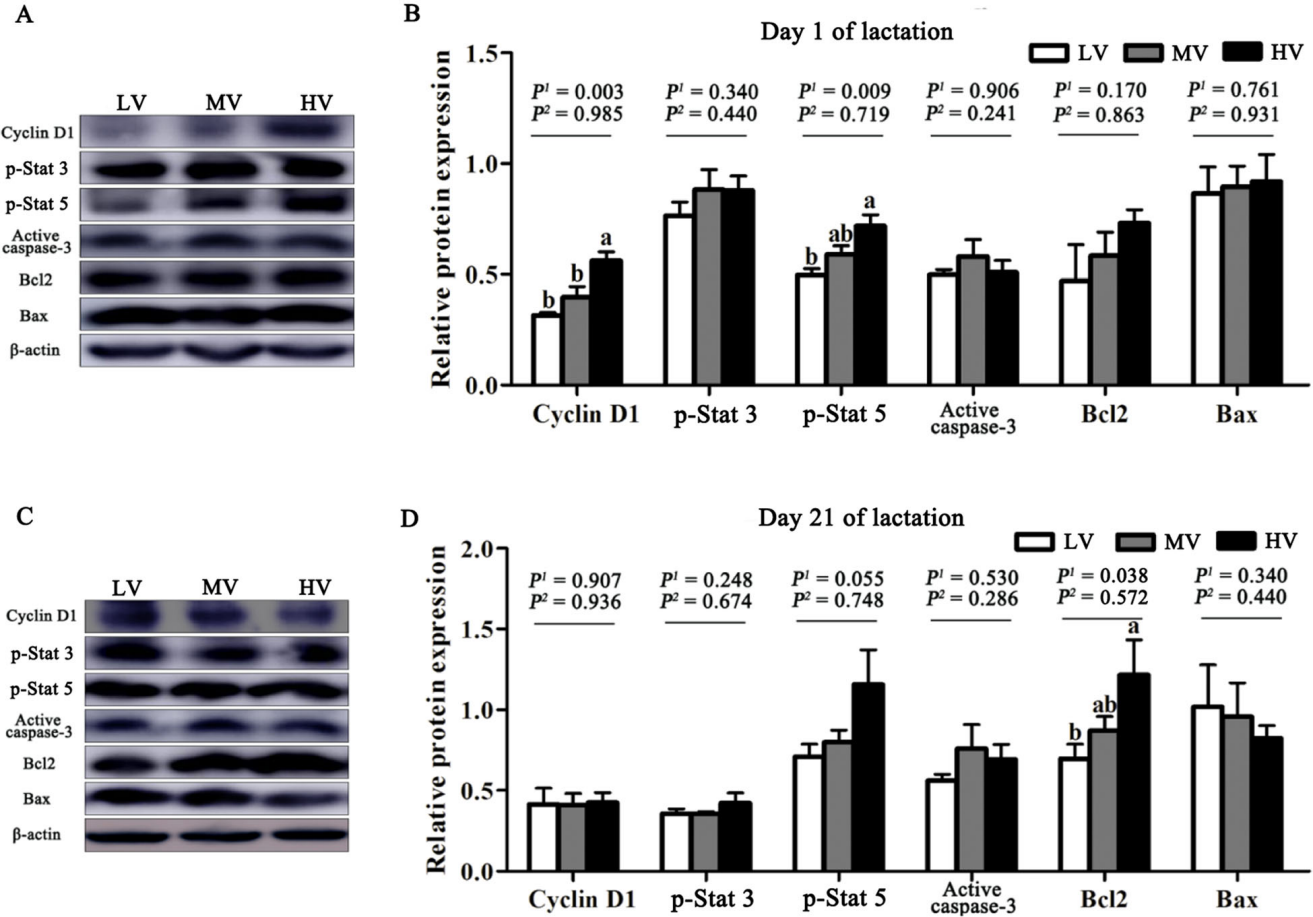
Effects of dietary valine to lysine ratio during late gestation on protein expression of cell proliferation, differentiation and apoptosis in mammary tissue. LV: total valine: lysine = 0.63: 1; MV: total valine: lysine = 0.73: 1; HV: total valine: lysine = 0.93: 1. **a**, **b** shows the relative protein expression in mammary tissue of gilts at day 1 of lactation, *n* = 4. **c**, **d** show the relative protein expression in mammary tissue of gilts at day 21 of lactation, *n* = 4. Cyclin D1: cell cycle protein D1; Stat: the signal transducer and activator of transcription; Bcl2: antiapoptotic B cell leukemia/lymphoma 2; Bax: Bcl-2 associated X protein. All data with error bars represent the mean ± SEM. *P*^*1*^ = linear, *P*^*2*^ = quadratic. Means not sharing the same letter are different (*P* < 0.05)

**Fig. 4:**
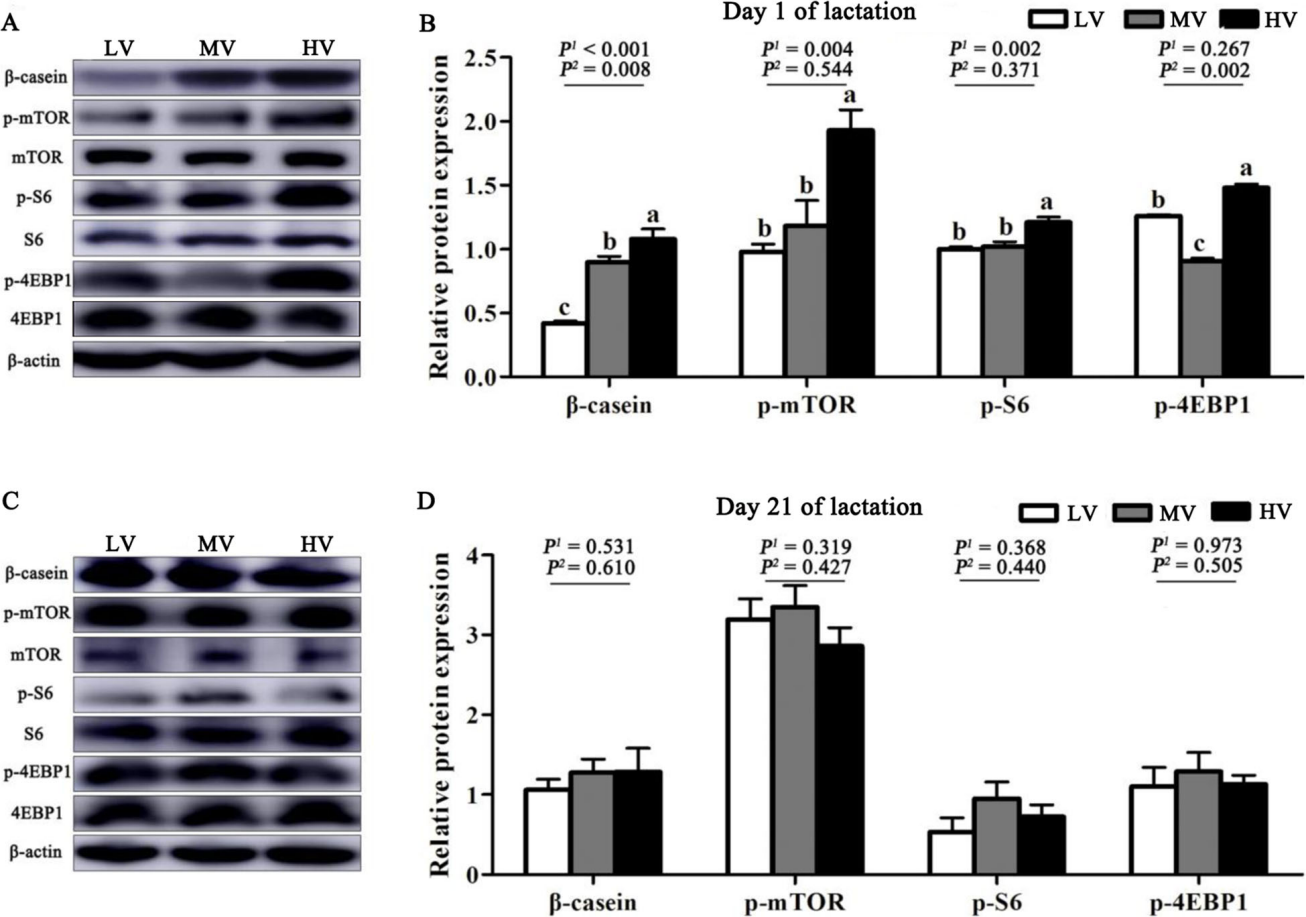
Effects of dietary valine to lysine ratio during late gestation on regulation of mTOR pathway in mammary tissue. LV: total valine: lysine = 0.63: 1; MV: total valine: lysine = 0.73: 1; HV: total valine: lysine = 0.93: 1. **a**, **b** shows the relative protein expression in mammary tissue of gilts at day 1 of lactation, *n* = 4. **c**, **d** shows the relative protein expression in mammary tissue of gilts at day 21 of lactation, *n* = 4. mTOR: mammalian target of rapamycin; S6: ribosomal protein S6 kinase; 4EBP1: 4E-binding protein 1. All data with error bars represent the mean ± SEM. *P*^*1*^ = linear, *P*^*2*^ = quadratic. Means not sharing the same letter are different (*P* < 0.05)

The morphological characteristics of the mammary tissue of gilts are presented in Fig. 1. The area of the lumen of alveolus of the mammary tissue was larger in the HV group than in the MV and LV groups (*P* < 0.05) at days 1 and 21 of lactation. As dietary valine increased, the expression of proteins related to BCAA metabolism (BCAT and BCKDH) linearly (*P* < 0.05) increased in the mammary tissues at day 1 of lactation (Fig. 2). Furthermore, maternal diet supplemented with valine during late gestation linearly increased the DNA, RNA, and total protein content in the mammary tissues (*P* < 0.05) at day 1 of lactation, and the highest content in the mammary tissue was observed when gilts were fed the HV diet (Table 8); however, the DNA and RNA content in the mammary tissues was not affected (*P* > 0.05) at day 21 of lactation (Table 8). The results in Fig. 3 indicated that valine supplementation did not change (*P* > 0.05) the protein expression levels of p-Stat3, active casepase-3, Bax and Bcl2 in the mammary tissues; however, dietary valine supplementation during late gestation linearly increased (*P* < 0.01) the expression of cyclin D1 and p-Stat5 at day 1 of lactation. Moreover, the expression levels of β-casein, p-mTOR and p-S6 linearly increased (*P* < 0.05) in the mammary tissue at day 1 of lactation with increasing dietary valine allowance, while there were no differences among three treatment groups at day 21 of lactation (Fig. 4).

**Table 8:** Effects of dietary valine: lysine ratio during late gestation on the content of DNA, RNA and total protein of mammary tissue

Items	Dietary treatments			Linear	Quadratic
	LV	MV	HV		
Day 1 of lactation					
DNA, μg/g	426.28 ± 75.33^b^	555.87 ± 42.85^b^	849.92 ± 102.88^a^	0.003	0.907
RNA, mg/g	2.57 ± 0.44^b^	4.31 ± 0.80^b^	6.93 ± 0.52^a^	< 0.001	0.710
Protein, mg/g	32.15 ± 6.04^b^	37.80 ± 0.75^ab^	47.65 ± 1.18^a^	0.013	0.917
DNA/Protein, μg/mg	13.47 ± 0.75	14.71 ± 1.08	17.95 ± 2.47	0.0742	0.901
Day 21 of lactation					
DNA, μg/g	656.77 ± 55.63	607.31 ± 68.66	817.06 ± 47.31	0.085	0.875
RNA, mg/g	4.89 ± 0.67	5.44 ± 0.70	5.58 ± 0.63	0.518	0.711
Protein, mg/g	40.89 ± 4.77^b^	44.62 ± 2.29^ab^	54.84 ± 2.86^a^	0.011	0.821
DNA/Protein, μg/mg	17.03 ± 1.42	14.68 ± 1.29	13.24 ± 1.64	0.890	0.781

Results were presented as mean values with their standard errors, *n* = 4. LV: total valine: lysine = 0.63: 1; MV: total valine: lysine = 0.73: 1; HV: total valine: lysine = 0.93: 1. Means not sharing the same letter are different (*P* < 0.05)

## Discussion

Adequate colostrum intake has been identified as one of the major factors that improve neonatal survival in pig production [17]. The total amount of colostrum intake per kg (birth weight) was positively associated with piglets’ survival, and it has potential long-term effects on the piglets’ daily weight gain until weaning [18, 19]. The crucial factors determining the synthesis of colostrum largely depend on the development of the mammary gland (e.g., number and efficiency of functional mammary epithelial cells) during late gestation [2]. Previous studies have shown that supplementation of valine increases the proliferation rate of porcine mammary epithelial cells in vitro and improves the milk protein and milk fat content in gilts [10, 11, 16], suggesting the importance of applying the nutritional approach to increase the mammary gland development and secretion of gilts. Therefore, we hypothesized that dietary valine supplementation during late gestation can increase the development of gilts mammary gland and improve milk production. In this study, we first observed that dietary valine supplementation to the gilts during late gestation significantly increased piglets’ performance; however, previous research has mainly focused on nutritional regulation of valine in lactation [20–22]. Notably, a linear increase in colostrum protein and fat synthesis was observed with the increase in dietary valine intake. The novel finding of the present study provides effective evidence that dietary valine supplementation during late gestation in gilts may increase mammary gland development or secretion capacity.

The development and secretion capacity of the mammary gland depends mainly on the number of mammary epithelial cells. The morphological characteristics, DNA, and RNA content in the mammary tissue is usually used as the main evaluation indices of mammary gland development [4, 23]. The alveolus is the basic milk secreting structure which consists of a single layer of mammary epithelial cells lining the alveolar lumen [2], and the area of lumen of the mammary tissue alveolus determines the number of mammary epithelial cells and lactation efficiency. In the present study, a greater area of the lumen of alveolus of mammary tissue in the group fed HV diet further supports the notion that dietary valine supplementation may improve the milk production. We also noted that the content of DNA and RNA in the mammary tissue was higher in the HV group than that in the LV and MV groups. A positive correlation has been demonstrated between mammary tissue DNA content and mammary epithelial cells proliferation in gilts [3]. Meanwhile, the RNA content in mammary tissue reflects cell activity. Similar to our study results, strong evidence from porcine mammary epithelial cells indicates that elevated extracellular concentrations of valine from 0.1 to 2 mmol/L increased cell proliferation [10]. In addition, the regulation of cell proliferation by BCAA has been demonstrated in cells, including hepatic cells [24], myocytes [25] and intestinal cells [26]. All these findings support the conclusion that valine promotes the mammary gland development in gilts; however, the molecular mechanism requires further investigation. Previous studies have reported that dietary amino acids exert a powerful influence on the level of plasma prolactin in lactation gilts [27, 28]. The results of present study revealed increased levels of plasma prolactin in lactating gilts supplemented with valine, suggesting it as a possible mediator of valine in promoting the development of the mammary gland. It is now clear that prolactin plays an important role in the activation of the Jak2/Stat5 signaling pathway, which regulates cell proliferation, cell differentiation and apoptosis to regulate milk production [29]. Signal transducer and activator of transcription 5 (Stat5) transduces extracellular amino acids signals to the nucleus of mammary epithelial cells and thereby regulates gene transcription during pregnancy, lactation, and weaning [30, 31]. In this study, the increased protein expression of p-Stat 5 in the mammary tissue of the HV group is probably indicative of enhanced milk production. In addition, it is noteworthy that a previous study indicated the important roles of mTOR in the regulation of protein synthesis, cell proliferation, and signaling pathway activation [32]. Valine is known to increase the phosphorylation of the mTOR signaling pathway [10]. Similarly, in this study, the phosphorylation of mTOR, 4EBP1 and S6 in the mammary tissues was significantly increased in gilts fed the HV diets during late gestation. Collectively, our data showed that valine may promote mammary epithelial cells proliferation via the activation of the mTOR signaling pathway. This is consistent with the *in vitro* data of our recent study [33], however, the molecular mechanism requires further investigation.

In addition, our results showed that the concentration of some amino acids in colostrum was increased by dietary valine supplementation. These results may be related to metabolism of valine in the mammary tissue. Valine catabolism begins with a transamination reaction catalyzed by the branched chain aminotransferases (BCATs) to form branched-chain α-ketoglutarate and glutamate [34], and then undergoes oxidative decarboxylation by the branched-chain α-keto acid dehydrogenase complex (BCKDH) [35]. In the present study, the expression of BCAT and BCKDH in the mammary tissue linearly increased with an increase in dietary valine supplementation at day 1 of lactation, which indicates that the catabolism of valine was increased. A previous study revealed that valine-derived glutamate is either amidated to generate glutamine or transaminated to synthesize alanine, aspartate, asparagine, proline, and polyamines [2]. Consistent with previous study results, free amino acid profiles in colostrum showed that glutamate and aspartate were increased in gilts fed HV diets during late gestation, as well as leucine, lysine and threonine. The amino acids in colostrum can be directly absorbed by suckling piglets, and have important physiological and nutritional functions for intestinal development of piglets [36, 37]. In contrast, the primary end product of valine metabolism is not complete oxidation; instead, it is beta-hydroxyisobutyrate, which is an ideal gluconeogenic substrate [38]. Therefore, valine may serve as a potential energy source for biosynthesis in the mammary tissue [38]. Further studies are needed to clarify the role of valine in mammary gland metabolism.

## Conclusions

In summary, the results of the present study showed that increasing the dietary valine to lysine ratio to 0.93 during late gestation significantly enhanced the piglet weight and average daily gain at weaning that was accompanied by increased maternal feed intake. The increased of prolactin concentration in plasma after valine supplementation may promote milk production. Dietary valine supplementation also resulted in the increased content of DNA, RNA, total protein content and the area of the lumen of alveolus in the mammary tissues that is probably linked to improved development of mammary gland.

## Data Availability

The datasets analyzed in the current study are available from the corresponding author on reasonable request.

## Abbreviations

4EBP1: 4E-binding protein 1
Bax: Bcl-2 associated X protein
BCAA: Branched-chain amino acid
BCAT: Branched-chain aminotransferase
BCKDH: Branched-chain α-keto acid dehydrogenase
Bcl2: Antiapoptotic B cell leukemia/lymphoma 2
Cyclin D1: Cell cycle protein D1
mTOR: Mammalian target of rapamycin
S6: Ribosomal protein S6 kinase
Stat: The signal transducer and activator of transcription
